# Who Invented the Possum? What Historians Can Learn from Disabled Innovation in Britain’s Responaut Communities

**DOI:** 10.1353/tech.2024.a920517

**Published:** 2024-01-01

**Authors:** Coreen McGuire

**Keywords:** disability, invention, hacking, polio, respiratory

## Abstract

This article provides a new exploration of disabled innovation that transforms our understanding of collective contributions to the history of science and technology. It does so by showing how a user network galvanized individual inventions into disabled expertise by tracking the development of two technologies—the Selectascan/Possum and the adapted Loudspeaking Telephone. Hamraie and Fritsch’s 2019 “Crip Technoscience Manifesto” defined “disabled expertise” by exploring how disabled technology modification has been devalued. This article takes up their manifesto’s challenge to combine disability history with science and technology studies by analyzing the technologies discussed in *Responaut*, a British quarterly magazine published between 1963 and 1989. Responauts were people who depended on respirators to breathe. This technological interdependence meant that users adapted an extraordinary variety of technologies to live well with respirators and modify their personal environment.

## Introduction

The term “respirator” is now often synonymous with ventilator. Between 1930 and 1980, it referred to an artificial or “mechanical respirator” for people with respiratory paralysis—a common consequence of polio.^1^ Responauts used and adapted an extraordinary variety of technologies to help them live well with respirators and modify their personal environment.^2^ A radical shift in perspective provided insights that enabled them to modify and invent new technologies, defined here as disabled innovation.

Disability historians have established disabled innovation as an important and recurring phenomenon, most recently in Virdi’s study of Andrew Gawley’s steel hands as the “physical, tangible representation of his experience.”^3^ Virdi shows that technologies do not simply represent experience; they are knowledge materialized. Historians of science and technology have long considered technologies “the objectification of knowledges and practices in new material forms,” yet they have only recently applied this insight to disability history.^4^ This is especially surprising given that science and technology studies (STS) scholars have long highlighted that users hack, tinker, consume, repurpose, and refuse to use technologies to enable a better fit with themselves and their preferences.^5^ Aimi Hamraie and Kelly Fritsch’s 2019 “Crip Technoscience Manifesto” identified this historiographical lacuna, elucidating how the particular kinds of knowledge developed by the disabled through object use and modification have been consistently commodified yet constantly unacknowledged.^6^ As critical disability studies scholars, Hamraie and Fritsch emphasize this expertise as the “skills, wisdom, resources, and hacks disabled people utilize for navigating and altering inaccessible worlds.”^7^ They pointed out STS scholarship’s limited engagement with disability studies, in part because of the social model’s explicit opposition to medical history. This article meets their manifesto’s challenge to combine disability history with STS by analyzing the technologies discussed in the quarterly magazine *Responaut*. It thus suggests that disability history analysis can subvert and complicate the stories of invention and discovery that have shaped the history of science.

Under the pseudonym Ann Armstrong, Doris Page (née Hayward) established *Responaut* in 1963. Armstrong had contracted respiratory polio in October 1955 and was placed in an iron lung in Prospect Park Isolation Hospital in Reading, United Kingdom, acutely ill and completely paralyzed (but for a toe), then moved to a specialist rehabilitation hospital the following year.^8^ The magazine’s initial subtitle, “a magazine for and by people using respirators,” changed in 1967 to “By, For and About Respirator-Aided and Other Gadget-Aided People,” to reflect its broader audience. This article looks at the 1963–67 issues for a narrow focus on respirator users and close reading. Armstrong features heavily, as she was the editor (along with notable guest editors) and wrote a memoir which contains further contextual source material.

Tracking the development of two remote switching devices with interpretative flexibility—the Selectascan and the Possum—shows how they facilitated telephone use, and British Telecom Archive material reveals the wider implications of these inventions.^9^ Individual inventions were galvanized by the user network centered around the magazine. This network was extended and strengthened by the Possum, which enabled fast typing, extensive correspondence, and extensive telephone conversations. Group usage of technologies allows a new exploration of invention and collaborative design in the context of relational and interdependent disability.^10^ If technologies are materialized knowledge, then we need to pay greater attention to how this knowledge moves between users to form an “innovation community.”^11^

Considering disabled innovation allows us to radically rethink scientific invention within communities working from the modern home. Household-based “thrifty” scientific practices creatively refashioning everyday objects have long been integral to shaping scientific epistemologies and methodologies.^12^ The historiography on these knowledge-making practices has focused on the early modern period—before boundaries emerged between instrument makers’ everyday collective experiments and specialist scientific knowledge characteristic of modern science.^13^ Presenting these boundaries as dichotomous reinforces an understanding that the nineteenth-century shift from home invention to institutional science was an important feature of modern science. Tracing how disabled people continually created and collaborated to rework domestic objects into the modern period, as well as following these inventions to and from the home and scientific institutions, challenges historical periodization and the normative standards of modern Western science.

### Inventing and Using the Possum and Selectascan

Switch systems like the Possum and the Selectascan enabled the remote operation of other devices. With very gentle pressure—just one muscular movement or exhale of breath—users could control other electrical equipment. They could thus manage their environment through technology, operating light switches, radios, telephones, and typewriters.

**Fig. 1:**
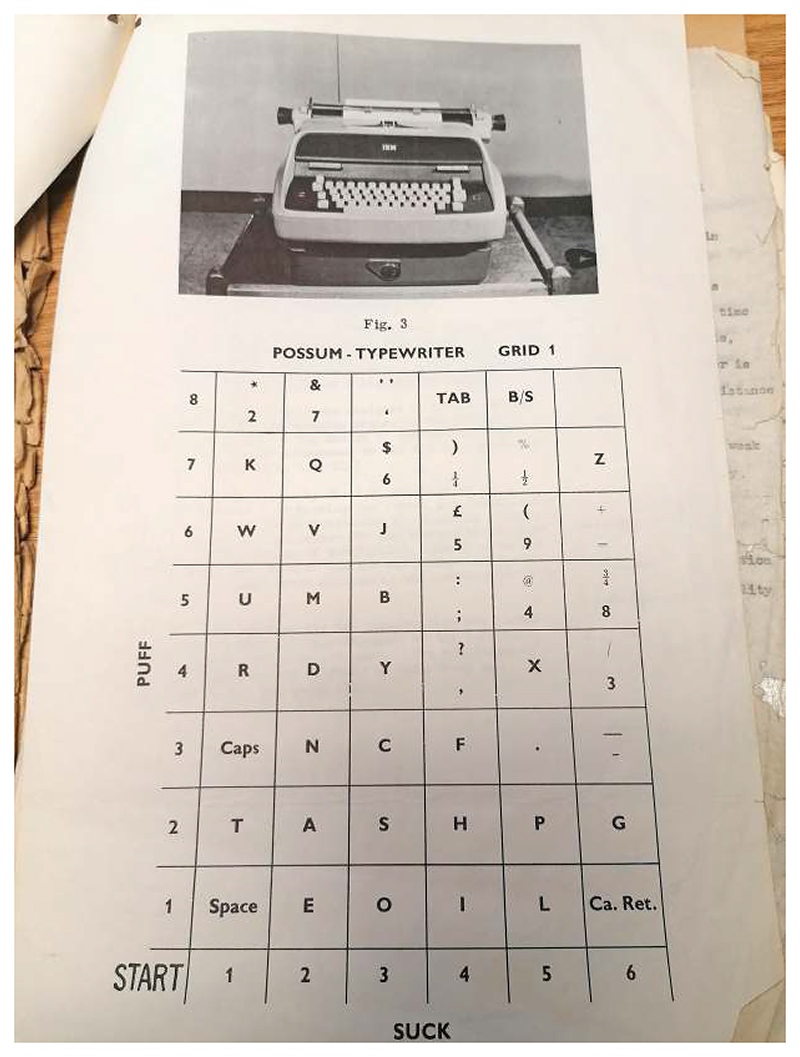
Grid Type. This grid replicated the keyboard keys and allowed users to navigate to the letter they wanted to type as audible clicks signaled the selection of a particular letter. The Possum switching system was linked with an IBM typewriter, which was sold at a reduced rate to those with respiratory paralysis. Although this system was very difficult to use at first, many users could type quickly and proficiently in this fashion, allowing them to work in a variety of professional roles. (Source: Letter Polio Research Fund C.R.A.D. to the Post Office, April 22, 1963, in “Telephone Concessions for the Completely Disabled,” 3, BTA.)

More than one technology, the Possum was “an electronic brain” that enabled the use of various technologies, applying the same principles as today’s breath-powered computer interfaces.^14^ Yet it was not easy to learn, especially with the electronic typewriter. One Possum user wrote, “I am struggling with this possum typewriter, at which it is necessary to huff and puff like mad, and if one huffs too madly or doesn’t puff quite madly quite enough then of course all sorts of things go wrong.”^15^ Another explained, “I sit with a little plastic tube in my lips and puff and suck in the form of a code. The first milestone of success came with the production of one triumphant word, ‘egg’ after two weeks of frustrated huffing, liberally laced with language I did not know that I knew.”^16^ This huffing allowed users to type via an electrical typewriter and grid system (see figure 1).

After activating the system by lightly sucking a tube, the user navigated across the grid by listening to audible clicks as the pointer moved; then, the clicks transmitted at a different frequency to allow the user to select the desired letter by moving down the columns.^17^ It took a huge amount of practice and repetition to perfect this skill. Contributor and Disablement Income Group activist Margaret Dixon Sussex described using it as a “wonder,” making it “possible for me to do, through one movement only—a nod of my head—an astonishing variety of things. I can operate two alarm bells, my respirator, television, radio, three different forms of heating and cooling, lights, and best of all the Loud Speaking Telephone.”^18^

Truly more than one technology, the principle behind this switching system was invented in two different institutional settings simultaneously. The Possum was developed in a formal setting by Reg Maling, a technician at Stoke Mandeville’s Electro-Mechanical Laboratory who helped people with spinal cord injuries. A husband-and-wife team embedded in the responaut community developed the Selectascan.

The magazine’s name connecting this community was inspired by the Cold War, which also shaped the technological references. Phenomenological studies of breathlessness emphasize that embodied possibilities are truncated or curtailed by breathlessness that leads to “bodily doubt” and a “loss of the familiar world.”^19^ Philosopher Havi Carel explains: “Breathlessness creates a new world, a new terrain to be navigated … what I once called a hill I now think of as a mountain.”^20^ *Responaut* supported those with respiratory paralysis to explore this new terrain through explicit association with cosmonauts and astronauts. The first issue explained *Responaut’s* name: “This useful coinage-of-a-word, like cosmonaut, indicates that we are charting new areas of experience and living. In this adventure we have—almost—everything still to learn.”^21^ Space metaphors in a later issue included the following comment:

Disability-wise, Responauts are living on the dark side of the moon. When you are firmly anchored to a mechanical respirator the comparative mobility, freedom and effectiveness of being able to get around the house in a wheelchair and use your own hands to do the ordinary everyday jobs seems highly desirable though unattainable.^22^

This comment conveys the widespread ignorance of the responauts’ plight and intimates their feelings of isolation, suggesting the restriction and immobility enforced by the artificial breath technologies linked them with space explorers.

Association with space travel was further cemented through the potential power of interstellar voyages to lead to the development of future technologies. Another *Responaut* contributor optimistically commented, “This is a very encouraging time for Responauts to be living, with astronauts and oceanauts being assisted by remote control, the efforts of other able bodied human beings and electronic equipment.”^23^ *Responaut’s* major focus on space was no doubt inspired by the iron lung, which greatly resembled a space ship with its visible rivets, shiny metal parts, and portholes.

Space metaphors provided a framework to relate to these objects and enact use. Armstrong meditated on this relationship when discussing the remote control system for operating home technologies with her feet. She explained, “With my control panel I identified strongly with astronauts. Like them I lived a restricted life in a tiny space and depended totally on machinery for my communications and air supply. How liberating to have the whole world at my feet!”^24^ The idea of an object “of safety and imprisonment” featured in Sussex’s editorial: “We are a greatly diverse group of people, with different problems, but most of us are fettered and entangled by mechanical aids of one kind or another. The curious thing is that, often, the more we are entangled by them, the more we are liberated.”^25^ Sussex’s quote illuminates the limited enablement associated with such objects and the diversity and heterogeneity of their users, reflective of the personal, subjective, and individual nature of breathing.^26^ Yet user-led space metaphors helped to create a common language and reference point that united the community around these necessarily individualistic experiences. Space imagery formed an “in-group” alternative to the popular public metaphors that associated iron lungs with steam engines to emphasize their strength and inhuman modernity.^27^ As well as offering hope, space metaphors could allow responauts to understand and accept new technologies in their homes.

Home technology modification was critical for those responauts like Armstrong who needed to work to remain at home rather than living in hospital. Because her husband had to work, her children were at school, and their relatives lived elsewhere, there was initially no way for Armstrong or other housewives in her situation to safely live at home. This was because there were no benefits or attendance allowance available to the “civilian disabled” who had not built up compensation benefits through working/insurance.^28^ Despite Armstrong’s repeated pleas to “go home to her children,” it took enormous effort and energy for her to return home, especially from her husband, who had to care for her fourteen hours a day while still working.^29^

The resulting inevitable financial strain of people living under such circumstances meant that being able to work was important for responauts, who complained bitterly of the financial difficulties that attended their lives at home. *Responaut* contributors advocated for a national disability income for all regardless of the cause of their disability. This advocation was the basis of a broader national campaign run by the Disablement Income Group, which often interacted with the *Responaut* and wrote about their movement, explaining that “at present, benefits depend upon how the injury or disease came about, and when. This is just nonsense, and anomalies arise.”^30^ As well as drawing comparisons with those disabled by war and industry, other contributors noted feeling “wistful on behalf of all the rest of us whose disabilities are due to germs and not compensated. I have to work jolly hard to pay for a housekeeper, expensive bungalow, large car etc. etc.”^31^ Some drew comparison to European countries to emphasize that Britain diverged from Europe in determining compensation on the source of the disability rather than its impact. This meant that those with industrial or wartime injuries were comparatively advantaged, especially in comparison to uninsured housewives, and other inconsistent situations arose. For instance, those undergoing treatment for respiratory tuberculosis received higher disability payments than other people with comparable respiratory illnesses, such as polio or pneumoconiosis.^32^

The *Responaut* contributors were also frustrated with the lack of state recognition of the importance of technologies to their lives; as one contributor emphasized, “My respirator, which I use every night and means *life* for me, may only consume a small amount of electricity, but it has to be paid for, but no special concession is made for this.”^33^ Another stressed this point by underlining that “gadget-aided man has arrived. Too many of us have spent the past decade learning to run our lives with mechanical help for it to be possible for authority to think of life-giving machinery as a luxury.”^34^ Technology was critical in enabling responauts to live at home with their family.

A later issue explicitly noted the important role of family, stating, “The only respos [*sic*] who can hope to live at home are those who have a large family with lots of members willing to help or a very rich family with the ability to buy in the necessary help or a very devoted family who will wear themselves to a standstill without giving up.”^35^ Many iron lung users were unable to live at home, and both their gender and their family played a substantial role in whether this was possible. One contributor complained of being put on a curfew as a man in an institution, whereas the married women who lay alongside him were able to go home freely (especially on weekends) because of their family support network.^36^ This example illuminates the extent to which disablement was moderated by networks of care and caring relationships and promotes an understanding of disability as *relational—that* is, how disability experiences were framed by family circumstances, and how disability affected family functioning in turn.^37^

This was the basis for the development of the Selectascan, the switching device that was invented by Bill Short while he was convalescing following a breakdown in his own health due to the stress of caring for his wife, Pat, who was in an iron lung.^38^ Bill was disabled by his care work. His invention was designed in close collaboration with his wife and with close attention to her needs and was entirely the result of the lack of care support he had, meaning that he needed her to be able to attend to herself without him. Pat described the moment of invention as a “discovery story”:

It is only two years since my husband gave up his job after a nervous breakdown. While he was convalescing, he had the idea of remote-control equipment operated by a slight pressure of the lips, slight movement of the finger, the voice, and—later on—small electric signals which are sent from the brain to any part of the body. Even though this equipment has saved my life mentally, and my husband wants to make its design and equipment his job, it seems that he is banging his head against a brick wall—except for several orders from a London hospital.^39^

The Selectascan was developed by Pat and Bill through collaborative work, which meant that Bill had a nearly complete understanding of her personal needs and a close intimation of her embodied knowledge. Breath itself was the main power source for this equipment, which was available for sale from W. H. Short, Avondale Engineering, Bolton, Lancashire.^40^ This is a modern example of scientific skill linked to “the collective knowledge of a family” rather than individual endeavor.^41^

When the Selectascan and the Possum were both available, *Responaut* contributors reviewed the merits of the user-driven Selectascan favorably in comparison to the Possum. For instance, one user wrote in to explain,

I have recently acquired a Selectascan—which is an electronic scanning device. This, in my opinion, is superior to the possum mainly because Selectascan can be operated by an acoustic switch. This obviates the need to have anything in the mouth, as is necessary when using a vacuum switch.^42^

However, after Pat Short died, Reg Maling and his project staff took over the maintenance of the Selectascan equipment and eventually absorbed it into the Possum project. Maling justified this by explaining in the *Responaut* that Bill wanted to forget about Pat’s paralysis and that such equipment was best served by those who could be “objective” scientists without emotional investment or lived experience of polio: “I should perhaps explain that none of the members of our project staff has any relatives with respiratory polio; this was quite deliberate, because we felt we could give best service if we were not in any way emotionally involved with the object of our work.”^43^

This example shows how the types of knowledge generated by the disabled and even their family members were not only devalued but also actively hidden by scientific standards prioritizing rational objectivity above and against embodied, sensorial, and emotional knowledge.^44^ Pat and Bill’s collaborative invention illustrates that binary categories collapse under the close scrutiny of knowledge-making practices. Historians of science have long emphasized the notion of objectivity as mobilized and reconceptualized to control and demarcate scientific knowledge production.^45^ Redefining objectivity as a marker of invention is especially relevant to technologies, which produce a particularly elevated “mechanical objectivity.”^46^

Maling’s letter about the Possum/Selectascan, in response to a complaint about the Possum, defended and established his primacy over the invention. He suggested that the magazine

would best aid the real needs of the disabled if care were taken to ensure that the facts of any complaint were checked before publication and also to ask the group or organisation being complained against to submit a reply for insertion in your journal at the same time as the original complaint.^47^

Further cementing Maling’s role as inventor of this switching technology was his collaboration with Paul Bates.

### Weaponizing the Telephone and Collaborating with the Post Office

Paul Bates contracted polio while on active service in Malaya in 1954, and so he received a war pension that he used in part to pay for his telephone account.^48^ He explained, “I could type, with a stick held in my mouth, and thanks to Reg Maling and his incredible brainchild—‘POSSUM’—I could, unaided, make and receive telephone calls.” He reasoned that “experience has shown that most disabled people can take their place in the working community and hold their own in competition with others if the occupation is chosen carefully.”^49^ This was also true for another Possum user who adapted it for use with a Dictaphone, allowing him to continue working as a lawyer. He emphasized that he had adapted the Possum beyond the scope of its original design

so that it comes into its own to an extent which I sometimes wonder whether even its creators envisaged—when, by the use of standard items such as telephones and intercom system, or the addition of special items such as a Dictaphone or type writer, it can make the most completely helpless substantially productive.^50^

This inventive telephone usage was not initially understood by the Post Office, which fully controlled the nationalized British telephone system from 1912 to 1981.^51^ Acting as an arm of the state, in the interwar years the Post Office was especially involved in the creation, modification, and maintenance of various technologies for the disabled, like the telephone service for the deaf.^52^ Following the establishment of the National Health Service and the Ministry of Health, Post Office engineers reduced the money and effort they put into such technologies and attempted to move the cost onto the Ministry of Health, which lacked the requisite technical expertise.^53^ Nevertheless, Post Office engineers worked with Reg Maling following a request from the Polio Research Fund charity in 1961 to amend their Loudspeaking Telephones circuitry so they could operate through the Possum controls, and they installed these adapted telephones in respirator users’ homes.^54^

However, the Post Office was not aware of the level and type of usage required until they brought in the subscriber trunk dialing system, enabling users to dial trunk calls without an operator but increasing local call charges. This was a catastrophe for users like Paul Bates, who immediately wrote to the Public Relations Office to protest the upcoming change and explain that he relied on the telephone “not only for emergency and essential purposes but also as a social outlet and a means of communication which I could not otherwise achieve.”^55^ The Public Relations Office explained that only local calls longer than three minutes would be charged at the higher rate. Bates responded that charging on a time basis would be “nothing short of disastrous” for those “who rely on the telephone so much as a social outlet” and hoped “that some concession will be made to those of us who in the past have found the telephone such a great weapon in the battle for trying to lead a normal life in spite of considerable physical disabilities.”^56^ After receiving little reassurance from the Post Office, he turned to his member of Parliament, emphasizing the entertainment and joy he associated with telephony. He stressed its role as a communication tool to both defend himself and organize his community: “I can honestly say it has been the greatest single weapon in the fight to lead a life approaching normality.”^57^ Bates’s anxiety resulted from “the mixed economy” of welfare characteristic of British care. This was reflected in his alternative demands for state aid and state support to work independently, exemplified in his plea for technologies that could turn “physically useless citizens into tax-payers.”^58^ Other responaut community members took up his cause and suggested group action to “make a united effort to make the Ministry of Health realise that a telephone for someone immobile and confined to one room is not a luxury, but a necessity.”^59^

Another responaut emphasized that the most important element of room design was access to electricity points and especially to telephony. The telephone system must be always accessible, ideally with more than one plug available. Users were happy to leave their emergency respirators in a cupboard, but the telephone always had to be at hand, and they explicitly wrote (echoing Armstrong) that they considered the telephone a “lifeline to the outside world, underlining my security.”^60^ This user’s “object of safety” was not their respirator but their telephone—the voice/breath object.^61^

The telephone allowed responauts to feel safe and connected to their family. It also allowed certain users to continue to work from home. This was especially important for disabled men, who could recover masculinity threatened by disablement through competing in the labor market and thus keeping their family (and care system) intact. This example provides an important corrective for narratives that have linked disablement to exclusion from the labor market, demonstrating that disabled people continued to work and provide for their families, in ways that may not have been obvious, or that may have been even purposefully hidden. For instance, Paul Bates used the telephone to “pass” as nondisabled over the phone. He explained that he used the Possum with the telephone to work and pass as able-bodied: “Through some freak of nature and in spite of my tracheotomy, I can speak irrespective of the phase of my respirator and this certainly helps to disguise my condition on the telephone.”^62^ He could therefore use his embodied knowledge of respirator technology to successfully sell the Egerton Stoke Mandeville Tilting and Turning Bed for its parent company, and he became its sales executive by the age of 33.^63^ Bates was also a “radio ham” (meaning that he used informal radio frequencies to transmit to other radio users), a popular way for responauts to communicate. One user emphasized its potential for overcoming race and class barriers: “There are no colour or class barriers in amateur radio and one never knows with whom one is going to talk to next.”^64^ Although the contributor did not specify his race, this suggests that work on *Responaut* could be used fruitfully in comparison with disability scholar Bess Williamson’s debate about U.S. responauts’ consumption of technologies to cement white middle-classness.^65^

Bates’s member of Parliament took up his case and wrote regularly to the postmaster general, arguing for a reduced rate. The member of Parliament explained that Bates required long conversations and emphasized the medical reasons that “apart from any other consideration, it is sometimes necessary for him and people like him to have a long pause for regaining their breath.”^66^ The Post Office’s continued refusal to reduce local call rates was based on the “principle of uniform charges” mandating that all customers were treated alike, so post, radio, telegraph, and television services were the same for all groups.^67^ Bates was quick to point out concessionary postal rates for the blind, but the postmaster general argued that the Post Office could physically check that the sender was blind but could not do the same for telephone users.

Bates continually advocated reduced charges by writing directly to the postmaster general, emphasizing that his disability was incurred while in service and that responauts used the telephone for their communication and occupation: “There are in fact some disabled who run a business from their bedsides and rely completely on the telephone for contact with their customers and suppliers.”^68^ Although the reduction for house-bound telephone users was not enacted, the director of the home counties (surrounding London) telecommunications branch did visit Bates to trial a faint speech amplifier telephone that amplified his voice to users on the other end of the line.^69^

Eventually, the Post Office collaborated with Reg Maling to provide a package set for respirator users to adapt their telephones to the Possum, though they were unwilling to commit until the Ministry of Health agreed to sponsorship.^70^ In 1962, they agreed to support a field trial of five devices sponsored by the Polio Research Fund and tacitly backed by the Ministry of Health.^71^ Reg Maling arranged for Stoke Mandeville to pay for the installation of Paul Bates’s Possum equipment and the rent (initially £7 10s. 6d. per quarter, then reduced to £3 10s. 0d., and finally to £ 1 10s. per annum), Maling having persuaded the Post Office to assess these charges on a no-profit, no-loss basis. Accessing the patchwork of services and mix of state, state-adjacent, and charitable bodies to ensure that Bates had the equipment he needed was difficult, even for privileged responauts like Bates, who used his military status and connections effectively.

On August 8, 1966, Mr. John Tilney told the Ministry of Health that Mr. Maling had to finance the Possum project from his savings.^72^ To legitimize the Possum project and shame the Ministry of Health into backing it, these debates frequently characterized Maling as a uniquely charitable and heroic inventor. The community knowledge network that had earlier shaped the Selectascan and Possum became obscure because the invention’s discovery was “fixed” as the collaborative enterprise “telescoped into an individual moment with an individual author.”^73^ In 1967, the Ministry of Health made the Possum equipment available through the National Health Service, and the original story of its invention is replicated on the Possum company website today.^74^

**Fig. 2:**
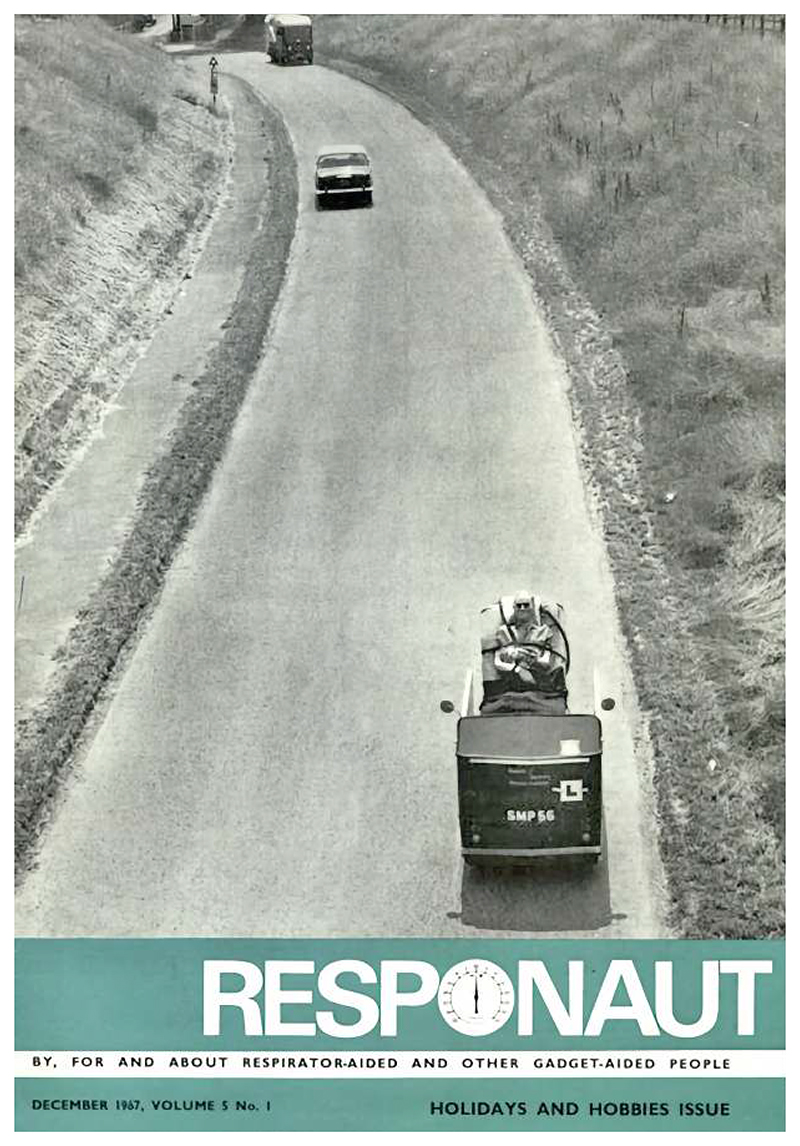
Driving Disabled Innovation. The 1967 cover of *Responaut* magazine shows Paul Bates grinning while driving his car, clearly inclined in his chair bed mounted on top of his battery-operated respirator. The dynamic image also represents Bates’s economic independence and the joy that technological innovation brought him. (Source: *Responaut 5*, no. 1 (1967).)

However, framing Reg Maling as someone who appropriated disabled knowledge without due recognition would be overly simplistic. Maling consistently highlighted the collaborative nature of the Possum project, underscoring that “it is to the severely disabled themselves that the major thanks must go; it was their courage in adversity which provided the determination to start the Project.”^75^ His collaboration and apparent friendship with Paul Bates were extensive and ambitious. Maling and Bates even worked together to integrate the Possum into a car’s controls, meaning that Bates could drive, having passed his test on September 7, 1967, after three months of practice.^76^ Bates could move the three fingers and thumb on his left hand; with his fingertips touching wire loops attached to switches, he could steer, accelerate, brake, and change direction. The car (see figure 2) was adapted to accommodate his chair bed and respirator, and the Possum selector allowed him to operate the emergency brake, horn, blinkers, and lights.

### Designing Gender Competence

The 1948 National Assistance Act allowed local health authorities to modify houses and provide appropriate aids such as the Possum.^77^ In practice, the services and facilities that the disabled could access depended on “geographical luck” and class status. This was pointed out in a House of Commons debate about National Health Service funding to produce the Possum when the initial Polio Research Fund Grant ended. Mr. Marsh Dickson, president of the Campaign for the Young Chronic Sick, noted, “It is interesting that the three Possum users are all of middle-class background with access to information from campaigns or voluntary organisations. My wife knew of these aids through the National Campaign for the Young Chronic Sick.” He wondered and worried, “How many people in North Kensington need these things? How are they to get to know about them—especially those who, because of lack of education or background, have no way of finding out for themselves?”^78^

This is reminiscent of Pat Short’s initial letter about the Selectascan, introducing the technology under the title “Finance Is the Problem.” Clearly, technologies were a crucial component of the typically mixed welfare that the social services provided for responauts in 1960s Britain. Prostheses could not compensate for comprehensive care and lack of income. Only with state support in place could users apply and creatively modify the range of devices discussed in *Responaut:* from lipstick to mouth sticks, automated painting boards to breath-controlled automobiles, radios to reading machines. More work is needed to fully understand user engagement with these devices, especially their reading practices.

Armstrong described using a reading machine with a button operated by her toe “as life-saving and inspiring mentally as my respirator was to me physically.”^79^ Using this machine made her think about other technologies that could be adapted to operate within the iron lung, and she described her initial imaginings of “other sorts of push-button machines that could be adapted to, or invented for, my needs,” recounting her designs to those around her.^80^

Armstrong gave a detailed account of how she used technologies in her fight to return to her husband and children at home in her 1985 memoir, *Breath of Life*. It included examples of abuse (though she did not describe it as such) from the hospital staff, including cutting skin from her dry lips, rubbing surgical spirit onto the friction burns on her shoulders, and spilling the spirit in her eyes (while she could not, of course, move her head).^81^ This overt abuse was compounded by a more general lack of care, resulting in a traumatic alienation from her body that she realized during her first physiotherapy session and vividly outlined:

Miss Clark lifted my right arm up straight, supporting it at the wrist and elbow. I looked up at her hands as she raised her arms and observed her holding this skeleton arm. I could see its bones—the humerus, ulna and radius—quite clearly through its tightly stretched skin. There was no other covering, no muscle, no fat, just the bare bones of an arm that would have been a gift to anyone studying anatomy. I saw the claws on the hand. They looked like the talons on the end of a chicken’s foot. The fingernails were humped and brown. Their overgrown ends curled completely over the tips of the fingers. The chicken-foot hand was covered with brown scales. Suddenly I realised with horror that it was my hand and my arm up there, being supported by Miss Clark.^82^

Compounding this realization was the comprehension that the staff had left her unclothed in the iron lung, so “the people who came up and looked in at me through the portholes could see this stark ugliness.”^83^ Armstrong describes this body alienation as a spur prompting her to overcome her “diffidence,” eat more, and concentrate on physiotherapy. She marked this new determination and resolve by asking her husband, Ken, to move the mirror on top of the iron lung so she could see her face and help him apply her lipstick. The importance of makeup is echoed in another *Responaut* contributor’s account of moving away from suicidal ideation after reading paperbacks from the hospital trolley:

It was one of those silly, stupid romances but I just read and read and wanted to read more and each time I was able to hold a bigger book. I felt I was in touch with life again, then the greatest joy was being able to clean my teeth myself and, after that, to be able to put on a little make-up.^84^

In both these cases, the application of makeup technologies comes at the narrative’s apex, marking the start of a campaign for recognition and care. Its important placement in these women’s accounts highlights the significance of makeup as a technology, to attract attention and demand recognition—”war paint” signaling the start of the fight. Makeup was especially important for women in respirators because selling cosmetics from home via the multilevel marketing scheme Avon was one of the ways they attempted to supplement gaps in their income.^85^

Yet this use of makeup falls outside the tired dichotomy of cosmetics as powerful versus cosmetics as patriarchal.^86^ The legal terms Armstrong used to secure support to live at home rested on her competence as a mother and wife—and disabled women have historically been either desexualized or represented as sexually dangerous.^87^ The fact that Armstrong was left naked while in the iron lung underlines this kind of desexualization. In addition, feminist disability scholars have shown that disabled people are frequently overpenalized for failing to perform gender appropriately.^88^ Makeup and other aesthetic technologies established appropriate gendered competence. The women who went home did so because their work as housekeeper and family manager was something that technological hacking allowed them to do from the iron lung. Armstrong explained that this could be managed by partitioning the home environment into designated areas:

The purpose-built home for a respirator-based mother must contain separate areas for the various functions presumed normal for a twentieth century woman. We are women of our time and peculiar to this decade, for without the invention of respirators we could not have survived—having survived, we have to push our position to its logical conclusion—that of adapting and making the best use of residual capabilities and talents, and so living at our most productive and satisfying level.^89^

Another contributor explained how to adapt the entire home environment with such functionality in mind—as well as cementing gendered work roles. She wrote,

A disabled mother must obviously be able to see what is going on in her kitchen; a father will need somewhere quiet in which to do his work where he can call his family if he needs them; a child will need to be in sight of its mother and siblings. … The home is the most important place for this fresh thinking in design to begin.^90^.

Highlighting the crucial importance of the housewife and mother’s work provided the moral and economic justification required to mobilize state support for these women to live at home. Indeed, the status of “housewife” was significant within the 1960s British social security system.^91^ As welfare state historian Gareth Millward explains in his history of the Disablement Income Group, unsurprisingly two “housewives” set up this campaign group because 1960s insurance-based compensation particularly discriminated against women who were less likely to work and, as legally “dependent upon the husband,” were not entitled to benefits while married or cohabiting.^92^ Even after the Labour Party’s victory in 1974 catalyzed non-insurance-based compensation, the Housewives Non-Contributory Invalidity Pension was delimited by a test establishing “whether a woman was capable of performing the ‘normal’ tasks associated with a homemaker.”^93^

The aesthetic and decorative control of electric technologies the Possum enabled was critical to the success of these “respirator mothers” (Williamson’s memorable term), and the Electrical Association for Women helped to allow this aesthetic electrification.^94^ Home design modification also enabled greater household control: one user described how from her bed she could see the kitchen reflected in a mirror, “and sliding doors into the sitting room make it possible for me to join in family pastimes or withdraw from them at will. I also control the television switch, which gives me considerable authority!”^95^ The same contributor suggested creative design modifications to doors and flooring, as well as explaining how to use mirrors, garden design, and light to increase “the distances over which the disabled person’s eye can travel during the day.” This was partly to ensure varied sensory stimulation and allowed responauts to enforce their personal taste and fashion sense onto their environment in a way that the writer noted gave them the same feeling of satisfaction “as if I were changing my dress.”^96^

Historian of science Graeme Gooday’s work shows that gender and class were crucial components for electricity’s initial acceptance in the home and eventual domestication. However, the gendering of “decorative electricity” impacted its reception and acceptance and mediated the understanding of expertise.^97^ Gendered epistemologies have long been used to delegitimize science and downplay the importance of minority contributors. Yet specifically women’s embodied knowledge was actively appropriated by British corporations and incorporated into important twentieth-century technologies.^98^

Interaction between gender and disability is crucial to understanding Armstrong’s struggle to be allowed to return home, not only entwined with gendered expectations but more broadly marked by the persistent epistemic injustice particular to the disabled.^99^ This was especially notable in her recollection of the experience of a nurse and doctor (both women) standing over her and questioning the potential provenance of scars on her ankles. When Armstrong (who was medically trained) explained that the scars were from the drip she had when her first baby was stillborn, the doctor advised the nurse, “She doesn’t know what she’s talking about,” without ever addressing her directly.^100^ This tendency to dismiss testimony—an example of what philosophers Carel and Kidd term “pathocentric epistemic injustice” (delegitimizing sick people’s knowledge claims)—colored the medical professionals’ assessment of her ability to care for herself at home.^101^ This epistemic injustice has resulted in failure to recognize the responaut community’s gadget hacking as disabled innovation.

In an article about switches, contributor Jim G. pointed out the collaboration elements in gadget hacking that disabled “thinking time” could facilitate:

Part of the fascination of creating an occupational gadget lies not only in the gadget but also in the enjoyment of the teamwork of “handicapped directo” and “engineer designer.” Thinking time supplied by the director can be exploited by the engineer and the product may well prove useful to other members of the handicapped fraternity.^102^

This personal embodied knowledge can be seen as individual disabled innovation, where the body/mind’s knowledge in particular circumstances shapes invention. Such individual innovation is clearly important for showing the friction between designer and user, implying that “disability things often defy the intention of their makers.”^103^ Sociologist and STS scholar Laura Maudlin has documented these processes in an important new project on how disabled folks and caregivers make their homes accessible.^104^

*Responaut’s* individual disabled expertise, however, was often necessarily shaped by collaboration, through either close family relationships or soliciting advice from the growing community of contributors. The role of “invisible technicians” was highlighted as particularly significant for group work processes, where “the tension between formal task descriptions and overt work on the one hand, and informal tasks and ‘behind the scenes’ work on the other, has been an important consideration.”^105^ Disabled users’ situated knowledge—their mulling, imagining, articulating—was collectively mobilized into design solutions and, indeed, scientific knowledge. Their embodied knowledge and practiced skills are useful for comparisons to the early modern artisanal knowledge that historians like Pamela Smith recognized as fundamental to the new scientific knowledge.^106^ Again we see that a long historical view of the history of science can help us legitimate the work of disabled users as genuinely scientific.

Arguably, there is a difference with individual disabled innovation if it is moderated by a group of users who thus transform it into disabled expertise. This is evident in direct questions. One reader noted, “I was interested to read of the prototype for the new respirator, I use a Cape engine, which has been very good, but I find the American Monaghan shell much more comfortable, better fitting and more free from draughts than the spiroshell,” and then asked, “Any more Respos find this?”^107^ Evidence of users trying out and alternating various “ ‘breath machines” emerges from discussions about how to circumvent the custom duties on specialist breathing equipment imported to Britain.^108^ Another contributor noted the need to experience such equipment through personal use, emphasizing that “most of us prefer our own breathing apparatus.”^109^ Shared common knowledge based on extremely individual experiences is in no way contradictory, and it is comparable to how artisanal knowledge was shared through the post.^110^ Historian of science Lea Biermann has identified that nineteenth-century craft knowledge did not depend on colocation and imitation, as American Postal Microscopical Club members transmitted locally produced knowledge about how to prepare microscope slides by posting slides alongside printed material, thus enabling the receiver to reverse engineer the technique for creating the slide.^111^

Similarly, *Responaut* contributors used the Possum to share new experiences of breathing equipment, in particular discussing the portability, sound, and attractiveness to facilitate leisure and communication. This adds to our understanding of the variability and heterogeneity of breath, also highlighting its importance in enabling such technological adaptation within an innovation community.^112^ That these users considered themselves a community is evident in an Australian writer’s response to news of another member’s death: “Living in each other’s pockets, we seem to make up a family and naturally we all feel his death as a loss.”^113^ Clare Jones’s edited collection shows that particular features of disabled community knowledge—altruism, aversion to patenting practices, and embodied ineffability—have historically made it vulnerable to commodification.^114^

## Conclusion

According to STS scholars Van Oost, Verhaegh, and Oudshoorn, “The dynamics of innovation processes that are initiated and shaped by a community of users” provide a fresh perspective on knowledge production in nonscientific settings.^115^ Or, in the words of Shaffer, if discoveries are defined through “ ‘artefacts constructed within research communities and as attributes granted to candidate events by the sanction of those communities,” then the Possum is part of a process of knowledge making created by a connected community of disabled users.^116^ Yet the public forgetting of Bill and Pat’s contributions exemplifies the erasure that often goes hand in hand with disabled innovation, as Williamson showed in the similar case of Sam and Betsy Farber, who collaborated to create the OXO grip line of kitchen tools.^117^ This erasure happens even though disabled innovation is predicated on using the individual’s body in experiments—historically a key criterion for inclusion within the scientific community as a marker of the heroic (male) inventor, for whom the “body as ‘field site’ is the ultimate heroic tale, in which the scientist potentially risks everything by engaging a battle against nature on his own internal territory,” according to feminist historian of science Naomi Oreskes.^118^

By thinking about objects as material “knowledge in transit,” this article has demonstrated that historical investigation of object knowledge can offset ahistoricism and reveal the importance of group disabled innovation in design. The design model of disability proposed by Bess Williamson and Elizabeth Guffey suggests that the experiences, meanings, and definitions of disability are shaped by design, and they explore disability “as a phenomenon that can be treated or ameliorated through digital or material things.”^119^ Selena Dyer, a historian of consumer culture, writes, “When approached as material culture, objects are more than witnesses to history, they are autonomous agents in the creation of that history.”^120^ Yet the objects we use only become meaningful through use. Tracking user innovation processes shows that disability history has never been “invisible” but has been actively hidden through epistemic systems that diminish and conceal it. As Sylvana Tomaselli’s work on women scientists illustrates, “collecting” historical examples of minority group members who succeeded in science risks perpetuating the idea that they contributed as “aids to science rather than its makers.”^121^

Answering “who invented the Possum” obscures the real epistemological significance of disabled innovation. Focus on appropriation distorts how knowledge networks collaborated and knowledge moved back and forth between homes and institutions. Just as examining vernacular knowledge and Western science through hybrid models rather than emphasizing one-way diffusion opens scientific spaces to reveal new historical actors, focusing on interaction collapses the distinction between disabled innovation and scientific theory.^122^ Western science’s dominance has rested on male standards of objective rationality that adapted flexibly to the normal working practices of those who dominated science at the time. The concept of disabled innovation challenges these standards. To quote Tomaselli again, “if we cease measuring contributions to science by the standards described above, if we cease to privilege individual achievement over collective work,” then we can subvert rather than perpetuate the epistemologies supporting them.^123^ Interdependence was an important component of disabled expertise.

As philosopher of science Michelle Gibbons recently pointed out, tracking a discovery to one eureka moment means that we are prioritizing results rather than processes and elevating the role of the individual mind over and above the technology.^124^ Asking who invented the Possum is the wrong question. Focusing on the technology prompts new questions: Why is the Possum still available, though the Selectascan is not? Why do we remember Reg Maling but not Bill and Pat and Ann and Jim and Paul? One answer is that the U.K. government sponsored Maling and funded the Possum device, leaving women like Pat without sufficient financial resources to survive at home. The obituary Ann wrote when her friend Pat died at the age of 34 emphasized the “appalling odds” Pat had faced in keeping her home; Ann also reprinted Pat’s (last) letter, which outlined her need for financial support following Bill’s struggle to care for her and their daughter while working at home selling the Selectascan. Ann pleaded for readers to remember Pat:

Those of us who have survived severe respiratory polio are still battling to return to the society to which we rightly belong. As long as I live there will be no veil drawn over the efforts of brave, cheerful, intelligent souls like Pat. We do not have such a surfeit of well-intentioned people in this world that we can afford the premature death of even one of them.^125^

Ann cautions that remembering is an active process. Remembering the Possum as a collaborative invention embeds disabled innovation in both the historical record and the historical object.

## Footnotes

1 These were different from modern-day ventilators, which use masks or more often breathing tubes in the windpipe or tracheostomy with unconscious users. McGuire, Virdi, and Hutton, “Respiratory Technologies and die Co-production of Breathing in the Twentieth Century,” 186.

2 Williamson, “Electric Moms and Quad Drivers.”

3 Virdi, “Material Traces of Disability.”

4 Suchman, “Working Relations of Technology Production and Use.”

5 Oudshoorn and Pinch, eds., *How Users Matter*, Oldenziel and Hård, *Consumers, Tinkerers, Rebels*; Von Hippel, “Dominant Role of Users in the Scientific Instrument Innovation Process”; Wyatt, “Non-users Also Matter.”

6 Hamraie and Fritsch, “Crip Technoscience Manifesto.” On commodity cultures: Jones, ed., *Rethinking Modern Prosthesis in Anglo-American Commodity Cultures, 1820-1939*.

7 Hamraie and Fritsch, “Crip Technoscience Manifesto,” 3. On broader disabled expertise in managing relationships and relationality: Hartblay, “Disability Expertise.”

8 Armstrong, *Breath of Life*.

9 Originally called Patient Operated Selector Mechanism (POSM), the acronym was expanded to correspond with the Latin word for “I can,” that is, Possum.

10 The term “relational” features in Curtis and Thompson, “Disability and the Family in South Wales Coalfield Society; 1920-1939.” The term “interdependence” is described by Turner and Blackie, “Disability and Political Activism in Industrialising Britain, c. 1830–1850,” 130.

11 Van Oost, Verhaegh, and Oudshoorn, “From Innovation Community to Community’ Innovation,” 183.

12 Werett, “Preserving Nature,” 320.

13 For a helpful review building on Shapin’s 1989 article on invisible technicians: Morus, “Invisible Technicians, Instrument-Makers and Artisans”; on household science relating to gender: Leong, *Recipes and Everyday Knowledge;* and in relation to gender and medicine: Fissell, “Introduction.”

14 This description comes from the 1964 film *Chairbound*, about rehabilitation practices at the Stoke Mandeville National Spinal Injuries Centre. The Possum clip begins at 3:33; https://player.bfi.org.uk/free/film/watch-chairbound-1964-online. /Another film featuring the Possum is on YouTube: https://www.youtube.com/watch?v=rn57ECJVSCAI.

15 G. W. Oxford, “Practising Patient,” *Responaut* 2, no. 2 (March 1965): 15.

16 “What They Have Done for Me,” *Responaut* 3, no. 4 (September 1966): 8–9.

17 Letter Polio Research Fund, C.R.A.D. to the Post Office, April 22, 1963, in “Telephone Concessions for the Completely Disabled,” 3, BTA.

18 Margaret Dixon Sussex, “This Freedom,” *Responaut* 3, no. 4 (September 1966): 1.

19 Carel, “Invisible Suffering.”

20 Carel, *Phenomenology of Illness*.

21 Ann Armstrong, “What It’s All About,” *Responaut* 1, no. 1 (December 1963): 2.

22 *Responaut 2, no. 2* (March 1965): 20.

23 *Responaut 2, no. 2* (March 1965): 22. Neil Armstrong was selected for NASA in 1962, and Doris Page announced her pseudonym in 1964, so it is possible, but unlikely, that her pseudonym was in homage to him.

24 Armstrong, *Breath of Life, 216*.

25 Margaret Dixon Sussex, “This Freedom,” *Responaut* 3, no. 4 (September 1966): 1.

26 Binnie, McGuire, and Carel, “Objects of Safety and Imprisonment”

27 Lawrence-Mackey, “‘Tron Lung’ as Metaphor.”

28 Millward, “Social Security Policy and the Early Disability Movement,” 281.

29 Armstrong, *Breath of Life, 231* postscript.

30 “D.I.G. This!,” *Responaut 2*, no. 3 (June 1965): 18.

31 *Responaut 2, no. 2* (March 1965): 10.

32 Lady Hamilton, “Integrating the Physically Handicapped,” *Responaut* 3, no. 4 (September 1966): 18.

33 Jean Heathcote, “Home-Help,” *Responaut*. 3, no. 3 (June 1966): 11.

34 *Responaut2, no*. 1 (January 1965): 1.

35 *Responaut 2, no. 2* (March 1965): 21.

36 “Respos on Pass,” *Responaut 2*, no. 3 (June 1965): 18.

37 Curtis and Thompson, “Disability and the Family.” Also McGuire, “Relational Disability and Invisible Illness in Industrial Britain.”

38 *Responaut* 1, no. 3 (June 1964): 14.

39 It is possible but not provable that the London hospital was Stoke Mandeville or connected to Stoke Mandeville (in Aylesbury). Pat Short, “Finance Is the Problem,” *Responaut I, no*. 3 (June 1964): 14.

40 “A Respo’s Equipment,” *Responaut* l, no. 4 (September 1964). 20.

41 Leong, “Collecting Knowledge for the Family,” 95.

42 “Accessory Equipment,” *Responaut* 1, no. 4 (September 1964): 21.

43 “Loophole in the National Health Service—Statutory and Voluntary,” *Responaut* 2, no. 2 (March 1965): 13-14.

44 Feminist historians of science outlined this point in relation to gender; on midwifery: Cody, “The Politics of Reproduction”; Jordanova, “Gender, Generation and Science.”

45 Oreskes, “Objectivity or Heroism?”

46 For the original point about mechanical objectivity: Daston and Galison, *Objectivity*; for the point about elevated epistemic authority: McGuire, *Measuring Difference, Numbering Normal*.

47 “Loophole in the National Health Service—Statutory and Voluntary,” *Responaut* 2, no. 2 (March 1965): 13-14.

48 Letter Paul Bates to the Postmaster General (hereafter PMG), “Subscriber Trunk Dialling System,” in “Concessionary Telephone Rentals for the Completely Disabled/Correspondence,” BTA.

49 Paul Bates, “Back to Work,” *Responaut* 3, no. 3 (June 1966): 11.

50 A Lawyer-Responaut, “The Value of Possum,” *Responaut* 4, no. 4 (September 1967): 4–5.

51 McGuire, “The Categorisation of Hearing Loss through Telephony in Inter-war Britain.”

52 McGuire, *Measuring Difference, Numbering Normal*.

53 For the case of the Medresco phone: McGuire, *Measuring Difference, Numbering Normal;* for discussion of wheelchairs: Woods and Watson, “In Pursuit of Standardization.”

54 Draft reply for the signature of the PMG to C. F. H. Gough MP, in “Concessionary Telephone Rentals for the Completely Disabled/Correspondence” BTA.

55 Letter Paul Bates to the Public Relations Officer, December 20,1960, in “Concessionary Telephone Rentals for the Completely Disabled/Correspondence.” BTA.

56 Letter Paul Bates to the Public Relations Officer P. A. Lock, January 20,1961, in “Concessionary Telephone Rentals for the Completely Disabled/Correspondence,” BTA.

57 Letter Paul Bates to Mr. Gough, January 23,1961, in “Concessionary Telephone Rentals for the Completely Disabled/Correspondence,” BTA.

58 *Responaut 3, no*. 3 (June 1966): 12.

59 John T. Prestwich, “Personally Speaking,” *Responaut* 4, no. 4 (1967); 1.

60 *Responaut 3, no*. 3 (June 1966): 14.

61 Binnie, McGuire, and Carel, “Objects of Safety and Imprisonment.”

62 Paul Bates, “Back to Work,” *Responaut* 3, no. 3 (June 1966): 11–12. For a full discussion: Brune and Wilson, *Disability and Passing*, 92.

63 “On the Road,” *Responaut* 5, no. 1 (December 1967): 4.

64 *Responaut 3, no*. 1 (September 1965): 4.

65 Williamson, “Electric Moms and Quad Drivers.”

66 Letter C. F. H. Gough to Reginald Bevins, PMG, July 24,1961, in “Concessionary Telephone Rentals for the Completely Disabled/Correspondence,” BTA.

67 Letter Reginald Bevins, PMG, to C. F. H. Gough, May 1961, in “Concessionary Telephone Rentals for the Completely Disabled/Correspondence,” BTA.

68 Letter Paul Bates to PMG, August 14,1961, in “Concessionary Telephone Rentals for the Completely Disabled/Correspondence,” BTA.

69 Memorandum from the Director, Home Counties Region (Telecommunications Branch), January 10, 1962, in “Concessionary Telephone Rentals for the Completely Disabled/Correspondence,” BTA.

70 The set comprised a modified Loudspeaking Telephone, a control rack incorporating a sender no. 1, a microphone on a 10-foot lead, and a transistor amplifier.

71 Discussed in a letter from H. G. Corpe to Mr. Maling, January 2 4,1962, in “Telephone Concessions for the Completely Disabled,”‘ BTA; and a letter from the Ministry of Health to Mr. Corpe, March 12,1962, in “Telephone Concessions for the Completely Disabled,” BTA.

72 “Chronically disabled (electronic aid),” HC Deb, August 8, 1966, vol. 733, ccl 173-89, Cl. 1176, https://api.parliament.uk/historic-hansard/commons/1966/aug/08/chronically-disabled-electronic-aid, HPD. Also “Patient-operated Selector Mechanism,” HC Deb, July 4,1966, vol 731, ccl6-7, https://api.parliament.uk/historic-hansard/commons/1966/jul/04/patient-operated-selector-mechanism #S5C V0731 P0_ 19660704 _HOC_109, HPD.

73 Schaffer, “Scientific Discoveries and the End of Natural Philosophy,” 397.

74 “Supply of Possum Equipment,” *Responaut* 4, no. 3 (1967). 20, also https://www.possum.co.uk/about-us/.

75 R. Maling, “Possum,” *Responaut 3*, no. 4 (1966): 6.

76 “On the Road,” *Responaut* 5, no. 1 (December 1967): 4.

77 “Housing and Home Helps,” *Responaut* 1, no. 3 (June 1964): 3.

78 North Kensington is an area of West London known for extreme inequalities along racial lines and the site of tire Grenfell Tower disaster in 2019. “Chronically Sick and Disabled Persons Bill,” HC Deb, December 5, 1969, vol. 792, ccl851-934, Cl. 1856, https://api.parliament.uk/lristoric-hansard/commons/1969/dec/05/chronically-sick-anddisabled-persons,HPD.

79 Armstrong, *Breath of Life*, 82.

80 Armstrong, *Breath of Life*, 101.

81 Armstrong, *Breath of Life, 66*.

82 Armstrong, *Breath of Life*, 82.

83 Armstrong, *Breath of Life*, 82.

84 Jill Forwood, “World of Women,” *Responaut* 4, no. 1 (December 1966): 26.

85 *Responaut2*,*no*. 1 (1964/65): 18.

86 Hornsey, “‘The Modern Way to Loveliness,’” 112.

87 On the institutionalization and segregation of (especially working-class) women regarded as sexually promiscuous and so intellectually disabled: Walmsley, ‘Women and the Mental Deficiency Act of 1913”; Goodman, “Pedagogy and Sex”; Jackson, *The Borderland of Imbecility*.

88 Cheng, “Sociological Theories of Disability, Gender, and Sexuality,” 115.

89 Ann Armstrong, “Patients’ Potential,” *Responaut* 1, no. 4 (1964): 5.

90 *Responaut 3, no*. 3 (June 1966): 14.

91 Millward, “Social Security Policy”; for more detailed analysis: Gulland, *Gender, Work, and Social Control*.

92 Millward, “Social Security’ Policy,” 282.

93 Millward, “Social Security’ Policy,” 292.

94 Williamson, “Electric Moms and Quad Drivers.”

95 *Responaut 3, no. 2* (September 1966): 14.

96 *Responaut 3, no. 2* (September 1966): 14.

97 *GooAay, Domesticating Electricity*, 174.

98 Rostvik, “Tampon Technology in Britain.”

99 Scully, ‘From “She Would Say That, Wouldn’t She?’ to ‘Does She Take Sugar?’”

100 Armstrong, *Breath of Life*, 122.

101 Kidd and Carel, “Epistemic Injustice in Healthcare.”

102 Jim G., “Switches, Switches, Switches,” *Responaut* 4, no. 4 (1967): 8-10.

103 Williamson, *Accessible America*, 9.

104 Laura Maudlin, Disability at Home, https://www.disabilityathome.org/

105 Star and Strauss, “Layers of Silence, Arenas of Voice,” 9. Also Shapin, “The Invisible Technician.”

106 Smith, Meyers, and Cook, eds., *Ways of Making and Knowing*; Smith and Beentjes, “Nature and Art, Making and Knowing,” 168,175.

107 *Responaut 2, no. 2* (March 1965): 18.

108 “Monaghan Breathing Equipment,” Responaut 2, no. 2 (March 1965): 17.

109 *Responaut 2*, no. 2 (March 1965): 18.

110 Beiermann, “‘A Method for Safe Transmission.’”

111 Beiermann, ‘“Method for Safe Transmission,’” 412.

112 Von Hippel, “Innovation by User Communities,” 82. Also Van Oost, Verhaegh, and Oudshoorn, “From Innovation Community.”

113 Letter from Helen Anderson, *Responaut* 4, no. 2 (March 1967): 23.

114 Jones, *Rethinking Modern Prosthesis*.

115 Van Oost, Verhaegh, and Oudshoorn, “From Innovation Community,” 183.

116 Schaffer, “Scientific Discoveries,” 388.

117 Williamson, “Getting a Grip,” 226.

118 Oreskes, “Objectivity or Heroism?,” 109.

119 Williamson and Guffey, *Making Disability Modern*, 5; Secord, “Knowledge in Transit.”

120 Dyer, “State of the Field,” 285.

121 Tomaselli, “Collecting Women,” 103.

122 Tilley, “Global Histories, Vernacular Science, and African Genealogies,” 113-14.

123 Tomaselli, “Collecting Women,” 103.

124 Gibbons, “Reassessing Discovery,” 69.

125 Ann Armstrong, “Life Enduring,” *Responaut 1, no*. 4 (1964): 1.
